# Endoscopic ultrasound-guided antegrade stenting combined with closure of the puncture route using self-assembling peptide solution in a jaundiced patient with ascites

**DOI:** 10.1055/a-2443-4069

**Published:** 2024-11-08

**Authors:** Hirotsugu Maruyama, Kojiro Tanoue, Tatsuya Kurokawa, Yoshinori Shimamoto, Yuki Ishikawa-Kakiya, Akira Higashimori, Yasuhiro Fujiwara

**Affiliations:** 1 12935 Gastroenterology, Osaka Metropolitan University Graduate School of Medicine School of Medicine, Osaka, Japan

Endoscopic ultrasound-guided hepaticogastrostomy (EUS-HGS) is not an established technique in patients with ascites. There have been reports of continuous ascites drainage 1 ; however, most cases have had poorly controlled ascites for a long time and there is a concern that fistula formation in the HGS route may be insufficient, leading to biliary peritonitis. Recently, the application of self-assembling peptide solution has been reported to reduce drain output and complications 2 . Aiming for simpler endoscopic therapy, we report an innovative method for EUS-guided antegrade stenting (EUS-AS) using a thin needle and closure of the puncture path with a self-assembling peptide solution.

A 55-year-old female patient with advanced gastric cancer who underwent gastrojejunostomy was referred because of jaundice. She was found to have obstructive jaundice due to lymph node metastases, moderate ascites, and obstruction of the distal side of the stomach on computed tomography scanning ( Fig. 1 ). Percutaneous transhepatic biliary drainage was therefore not appropriate. Although there was ascites present between the stomach and liver ( Fig. 2 ), we decided to try EUS-AS using a 22G needle.

**Fig. 1:**
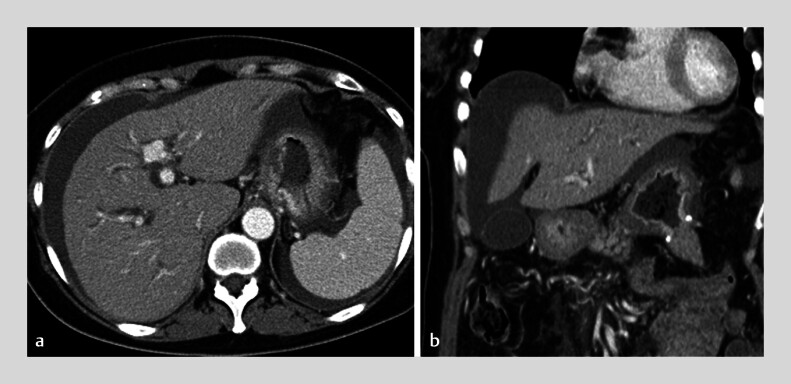
Preoperative computed tomography scan showing ascites present on the surface of the liver and between the stomach and liver.

**Fig. 2:**
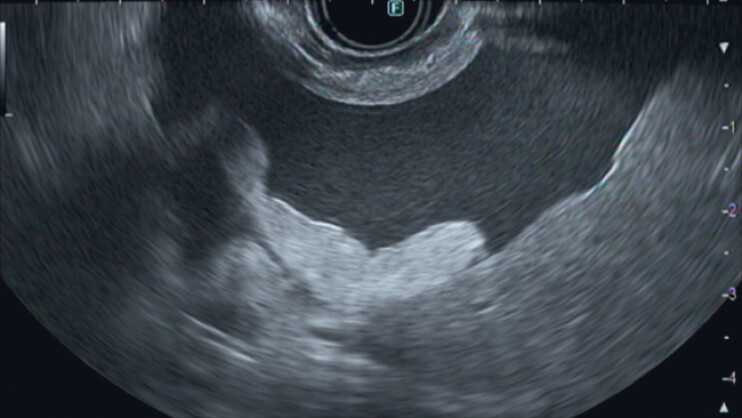
Endoscopic ultrasound images taken from the stomach showing moderate ascites between the stomach and the liver.

We performed the preloading guidewire method 3 , advancing the guidewire into the duodenum after placing double guidewires using an uneven double-lumen cannula (UDLC; Piolax, Tokyo, Japan). We confirmed the site of the stricture within the bile duct and deployed an uncovered self-expandable metal stent (Yabusame Neo; Kaneka). Finally, we applied 5 ml of self-assembling peptide solution into the puncture route using the double-lumen cannula ( Video 1 ). Although we did not place a stent into the puncture route, there were no adverse events ( Fig. 3 ).

An endoscopic ultrasound-guided antegrade stenting procedure is combined with closure of the puncture route using a self-assembling peptide solution.Video 1

**Fig. 3:**
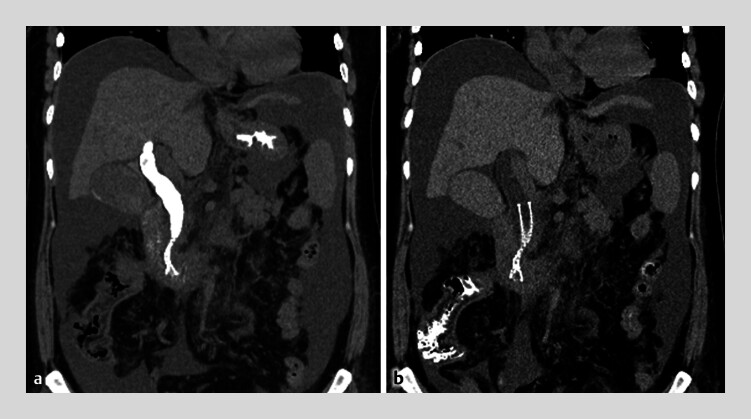
Postoperative computed tomography scan showing: **a** no evidence of leakage of contrast medium immediately after the endoscopic procedure; **b** the disappearance of contrast medium from the bile duct the following day.

This method is simple and appears to effectively avoid the associated adverse events without requiring stent placement into the HGS route, thereby shortening the procedure time, reducing the number of dilation procedures, and reducing the stress for the endoscopist. The self-assembling peptide solution is simple to apply and it can easily be done by anyone.

Endoscopy_UCTN_Code_TTT_1AS_2AD

## Notes

Endoscopy E-Videos https://eref.thieme.de/e-videos
*E-Videos* is an open access online section of the journal *Endoscopy* , reporting on interesting cases and new techniques in gastroenterological endoscopy. All papers include a high-quality video and are published with a Creative Commons CC-BY license. Endoscopy E-Videos qualify for HINARI discounts and waivers and eligibility is automatically checked during the submission process. We grant 100% waivers to articles whose corresponding authors are based in Group A countries and 50% waivers to those who are based in Group B countries as classified by Research4Life (see: https://www.research4life.org/access/eligibility/ ). This section has its own submission website at https://mc.manuscriptcentral.com/e-videos .
